# Forest biomass change estimated from height change in interferometric SAR height models

**DOI:** 10.1186/s13021-014-0005-2

**Published:** 2014-09-10

**Authors:** Svein Solberg, Erik Næsset, Terje Gobakken, Ole-Martin Bollandsås

**Affiliations:** 1 Norwegian Forest and Landscape Institute, Ås 1431 Norway; 2 Norwegian University of Life Sciences, Ås 1432 Norway

**Keywords:** Forest monitoring, Biomass, Carbon, InSAR

## Abstract

**Background:**

There is a need for new satellite remote sensing methods for monitoring tropical forest carbon stocks. Advanced RADAR instruments on board satellites can contribute with novel methods. RADARs can see through clouds, and furthermore, by applying stereo RADAR imaging we can measure forest height and its changes. Such height changes are related to carbon stock changes in the biomass. We here apply data from the current Tandem-X satellite mission, where two RADAR equipped satellites go in close formation providing stereo imaging. We combine that with similar data acquired with one of the space shuttles in the year 2000, i.e. the so-called SRTM mission. We derive height information from a RADAR image pair using a method called interferometry.

**Results:**

We demonstrate an approach for REDD based on interferometry data from a boreal forest in Norway. We fitted a model to the data where above-ground biomass in the forest increases with 15 t/ha for every m increase of the height of the RADAR echo. When the RADAR echo is at the ground the estimated biomass is zero, and when it is 20 m above the ground the estimated above-ground biomass is 300 t/ha. Using this model we obtained fairly accurate estimates of biomass changes from 2000 to 2011. For 200 m^2^ plots we obtained an accuracy of 65 t/ha, which corresponds to 50% of the mean above-ground biomass value. We also demonstrate that this method can be applied without having accurate terrain heights and without having former in-situ biomass data, both of which are generally lacking in tropical countries. The gain in accuracy was marginal when we included such data in the estimation. Finally, we demonstrate that logging and other biomass changes can be accurately mapped. A biomass change map based on interferometry corresponded well to a very accurate map derived from repeated scanning with airborne laser.

**Conclusions:**

Satellite based, stereo imaging with advanced RADAR instruments appears to be a promising method for REDD. Interferometric processing of the RADAR data provides maps of forest height changes from which we can estimate temporal changes in biomass and carbon.

**Electronic supplementary material:**

The online version of this article (doi:10.1186/s13021-014-0005-2) contains supplementary material, which is available to authorized users.

## Background

Management of forest carbon (C) stocks is increasingly addressed due to its impact on the global greenhouse gas cycle and climate. Deforestation contributes to a significant fraction of the total anthropogenic C emissions [1],[2]. The C loss from land use change, i.e. mainly deforestation, is currently about 900 Mt/yr [2]. C loss from forest damage and mortality is negligible in comparison, estimated to be only 1.9 Mt/yr for disturbances in the Amazon [3], and only 13.5 Mt/yr in the extremely extensive mountain pine beetle epidemic in British Columbia [4]. The suite of methods for mapping, monitoring and estimating parts of the forest C cycle is expanding rapidly, including field inventory, modelling and remote sensing. Field inventory is widely used for national forest inventories, and its feasibility for monitoring C stocks in forests such as the Niassa National Reserve miombo woodland in Mozambique has recently been demonstrated [5]. Remote sensing has a wide range of applications. For example, time-series of vegetation indices from MODIS has been used to estimate carbon fluxes in Alaskan ecosystems during 2000-2010 [6] and sustainable amounts of wood harvesting in Southeast Asia [7]. Models such as the Forest Vegetation Simulator (FVS) can be used to compare effects of forest management alternatives, e.g. tree species selection, on future C sequestration [8].

Deforestation in the tropics is of particular significance due to its rapid speed [9], and the REDD (Reducing Emissions from Deforestation and Forest Degradation in Developing Countries) initiative aims at reducing the C losses through performance-based credits by comparison of performance against a business-as-usual reference emission level. In addition to deforestation, forest degradation and enhancement of carbon stock through forest growth are other components of the forest C changes that should influence the REDD credits. In order to realize this payment-for-ecosystem-service, the tropical countries need to document their annual changes in forest C stocks, and satellite remote sensing is likely to be a major data provider for this [10],[11]. Such data would also enable detection of logging areas for possible counteractions. However, there is a need for new satellite remote sensing methods for REDD. Firstly, there is a need for methods that can overcome the limitations of today’s methods, i.e. clouds, small areal coverage and failure to detect C stock changes other than deforestation. Secondly, there is a need for historical data on forest changes for the business-as-usual emission level.

Optical satellite data is the dominating remote sensing method today, having the limitation that the correlation with biomass is weak and tend to saturate at low levels. The PRODES project in Brazil represents state-of-the-art [12]. Annual, full-coverage of Brazil is obtained with about 233 Landsat images, from which clear-cuts are detected from a semi-automatic pixel-unmixing classification based on soil and shadow fractions. Although this is carried out in the most cloud-free season of August-September, clouds are preventing data in some areas. Persistent cloud cover is common in tropical forest areas [13]. A second limitation with the method is that it merely tracks land cover changes such as changes from forest to non-forest and vice versa that can be detected, while forest degradation is hardly detectable, and they make up a considerable share of cuttings in the tropics. Finally, the conversion of the annual clear-cut area into changes in forest C stocks is crudely obtained by using fixed emission factors. The recently upgraded Global Forest Watch [14] is a new and valuable forest monitoring tool; however, it is also largely based on Landsat [15] and is apparently having the same abilities and limitations as the PRODES system.

Remote sensing methods that provide 3D data have a considerable advantage in comparison with 2D, optical data. They provide measurements of forest height, and its changes, which is crucial for forest biomass changes. Airborne 3D remote sensing, i.e. airborne laser scanning (ALS) or stereo photogrammetry, is a more accurate tool than optical satellite data and it can detect also forest degradation and growth [16]. For most countries, applications with complete ALS coverage are cost-prohibitive. The feasible application of ALS would be strip sampling, which may provide accurate estimates of C stocks changes compared to other methods [17]. In [18], carbon stocks were estimated with 1-ha resolution at the national scale of Panama, based on a sample of field plots and ALS in combination with full areal coverage of Landsat and MODIS data. The accuracy was estimated to 20.5 t/ha of C at the 1-ha pixel level.

The current study is focusing on interferometric SAR (InSAR), which can provide 3D data. Methods based on satellite SAR (Synthetic Aperture RADAR) are getting more attention, and they may resolve the limitations with optical data. SAR is an imaging RADAR system. The cloud problem is non-existent with SAR, because the longer wavelengths, commonly 3-70 cm, penetrate clouds. In Sweden, clear-cuts have been accurately detected as a decrease in the backscatter of ALOS PALSAR [19]. The idea we pursue here is based on changes in surface height obtained from InSAR, where changes in biomass and carbon stocks can be retrieved directly from changes in InSAR height. Logging leads to a reduction in height, while forest growth leads to an increase in height. Heights can be derived from SAR data in two or more ways, mainly by phase differences (interferometric SAR, InSAR) or by parallaxes (radargrammetry) in a SAR image pair. These techniques go back to the 1960s and 1970s, when they were demonstrated with stereo acquisitions in airborne SAR systems [20],[21].

With the satellite based InSAR method the heights are derived from phase differences between two SAR images taken from different positions in space. This can provide accurate height measurements; however, the accuracy depends on various acquisition properties. In particular for short wavelength SAR (e.g. X-band) over forested areas the SAR imaging needs to be carried out by two satellites going together in a close formation. This is called a bi-static, or single pass, acquisition, where one satellite is submitting microwave RADAR pulses and both satellites receive the same echoes from Earth’s surface. A bi-static acquisition removes temporal de-correlation, i.e. phase noise caused by differences in the position of branches and in moisture. Phase noise is also influenced by the distance between the satellites, i.e. the baseline, which should neither be too large nor too small. With increasing baseline there is an increasing volume de-correlation caused by an increasing difference in the look angle (local incidence angle) into the canopy volume. Contrary to this, when the baseline is very small the noise increases because of quantization errors, i.e. a given height correspond to a tiny fraction of a 2π cycle of phase difference. In order to compress data onboard the satellite the data is typically compressed to 5 or 8 bit [22], and a tiny fraction of such a number will correspond to a crude height measurement. Finally, random errors and phase noise will be relatively large when the backscatter signal is weak, i.e. a low signal-to-noise ratio, depending on incidence angle, polarization and topography [23].

It has recently been demonstrated that forest biomass, or the equivalent stem volume, is strongly related to InSAR height, i.e. the height above ground of the center of the SAR echo [24]–[28] This relationship may vary with stand structure, in particular tree number density [29],[30]. However, existing studies show fairly accurate models without taking forest structure into account, likely because the variation in forest structure is moderate, e.g. [24]. In addition, it has been demonstrated that loggings, i.e. clear-cutting and forest degradation, can be detected as temporal changes in surface height [31],[32]. The latter studies detected the logging-induced decreases from the 90 m SRTM C-band DSM to a recent and higher resolution X-band DSM.

One significant step further from detection of logged areas would be to estimate the corresponding changes in above ground biomass (AGB), or C stocks, as surface height changes over time. This should cover not only deforestation, but also forest degradation and forest gain and growth. In addition, the 11 year InSAR height changes from SRTM in 2000 to Tandem-X in 2011 could be used for the business-as-usual reference levels. The C-band SRTM had a near-global coverage and Tandem-X DSM has a global coverage. Hence, these two surface models can be combined and used to detect surface height changes in forests, and possibly estimate changes in forest C stocks. Besides possibly providing estimates in C stocks, this would also provide maps showing the changes, i.e. both decreases from logging and increased from forest growth.

A main question is how accurate C stock, or AGB changes can be estimated by means of InSAR, and in addition, there are several possible limitations, including:

1. The lack of a digital terrain model (DTM) making it impossible to derive AGB and C stocks,
2. The difference in SAR wavelength between the C-band SRTM and the X-band Tandem-X,
3. The coarse resolution of the C-band SRTM (90 m),
4. The lack of AGB data from field from the time of the SRTM acquisition, and
5. Unstable relationships between AGB and InSAR height.

Crucial here is that it is the changes in C stocks we are after, and not the C stocks themselves. Hence, the lack of a DTM could be overcome if AGB and InSAR height are proportional, i.e. a straight linear relationship without saturation going through origin. This has been found to be the case for a tropical forest in Brazil [33] and for a spruce forest in Norway [24]. Also, without a DTM the relationship between AGB and InSAR height needs to be available from an external model. In the present study an external model was fit by processing the Tandem-X data against a DTM and estimating the relationship between AGB and InSAR height for a number of field plots.

The main objective of this study was to estimate how accurately AGB changes (ΔAGB) over time can be estimated from DSM changes between the SRTM C-band DSM acquired in 2000 and a Tandem-X DSM from 2011. The specific aims were to estimate

1. bias, accuracy and precision for ΔAGB on 200 m^2^ plots, and
2. the accuracy in the spatial variation in ΔAGB over the study area.

In addition, we aimed at quantifying how much the performance of this method is reduced

3) because different SAR wavelengths are used at the two points of time (C-band in 2000 and X-band in 2011), and
4) if plot biomass values were not available to fit models at the first point of time (year 2000) and if a DTM was not available (which would be the general case for most REDD countries).

We had at hand a unique data set for this study, comprising repeated field inventory, repeated airborne laser scanning, and full coverage X- and C-band SRTM and Tandem-X data.

## Results and discussion

### AGB — InSAR height relationship

**Figure 1:**
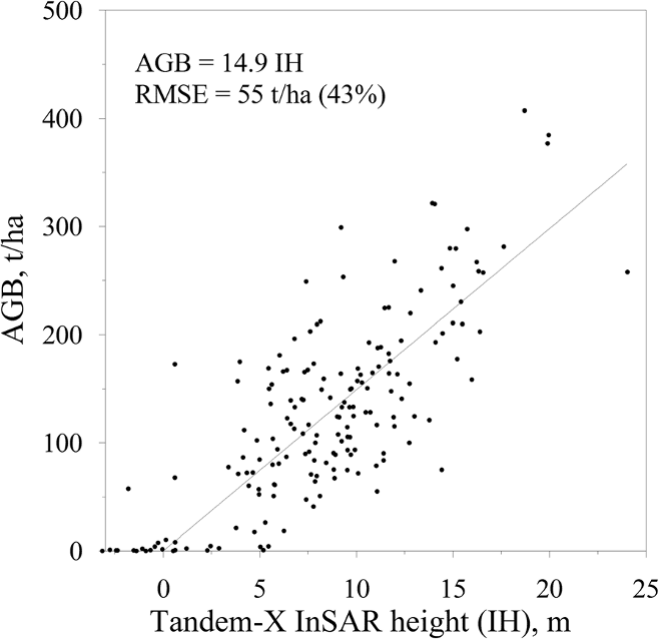
**Relationship between AGB and InSAR height from Tandem-X for 200 m** ^**2**^ **plots, fitted with a no-intercept regression model.**

The relationship between AGB and Tandem-X InSAR height could be represented as a proportionality, where AGB inceased by 14.9 t/ha per m increase in InSAR height. We first fitted an ordinary linear regression model, which provided an estimated intercept of 16 t/ha and a slope of 13.4 t/ha/m, and had a RMSE of 54.3 t/ha. The intercept was negligible and not statistically significant from zero. We re-fitted the model without an intercept, as shown in Figure 1. The exclusion of the intercept had nearly no effect on the model performance, i.e. the RMSE showed almost no increase.

A positive correlation between biomass and InSAR height is as expected, because they both increase with tree height and stand density. In a theoretical approach [29] demonstrated that the relationship between stem volume, or correspondingly biomass (B), and height (H) follows a power law function:

1 $$\left[7\right]\;B\propto H^{a}\text{.}$$

If the height variable, H, represents mean tree height or top height the *α* takes a value in the range 1.5-2.0 [29],[30],[34], which implies a curvilinear relationship with a saturation effect for high biomass values. In the present study the height, H, is not representing tree height, but rather canopy height. InSAR height, as any type of canopy height variables, depends not only on tree height, but also on the shape of each tree crown and on stand density (the amount of gaps). Proportionality between biomass and InSAR height, or at least *α* values close to unity, has been demonstrated both in boreal and tropical forests [24],[25],[33].

A few outliers having zero InSAR height and AGB > 50 t/ha might be observations where logging has occurred between the field inventory in 2010 and the Tandem-X acquisition in 2011. Stand structure might affect the relationship between biomass and InSAR height [29], and further studies may reveal whether this is the case. It is unlikely that more accurate biomass estimates could be obtained for these plots being as small as 200 m^2^, taking into account what is achieved with other remote sensing methods including airborne laser scanning [35]. Hence, the potential to improve the accuracy taking into account stand structure seems limited.

Some plots had negative InSAR heights, down to -3 m. This is attributable to various types of errors in the Tandem-X DSM. These error types include remaining bias and ramp errors over the entire study area, phase discontinuities in steep topography such as forest stand edges, and remaining residual errors not removed by the multilooking and Goldstein filtering (see below).

We add here that in a real case in a tropical country without a full coverage DTM the model would have to be obtained in another way than here, e.g. from plots in some smaller study area with an accurate DTM or from scattered field plots in a sample survey having accurate terrain heights from GPS measurements on the plots.

### Accuracy in estimated AGB changes at plot level

**Figure 2:**
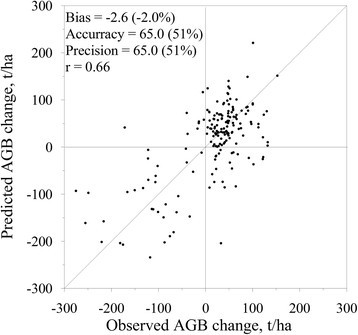
**Predicted AGB changes for 200 m**^**2**^**plots based on InSAR height changes from the SRTM C-band DSM and a Tandem-X DSM, and the model AGB = 14.9IH, where IH is InSAR height above ground.** Bias, accuracy and precision are given in t/ha as well as in % of mean biomass in 2010 (127 t/ha).

The obtained model (Figure 1) was used to predict AGB changes on the field plots from 2000 to 2011 directly from DSM changes. These predictions were clearly correlated to field measured AGB changes with r = 0.69 (Figure 2). The RMSE of these predictions was 67 t/ha, which was only slightly higher than the obtained accuracy, 59 t/ha, in the model in Figure 1. The negative changes seen in Figure 2 are mostly large and represent logging, while the positive changes are smaller and results from forest growth.

### The influence of SRTM band and separate models at two points of time

The relationship between AGB and InSAR height was fairly similar with Tandem-X in 2011 and the X- and C-band SRTM in 2000 (Table 1). After correcting vertical offset and ramp errors in the SRTM DSMs based on ground control points, and subtracting the DTM, we obtained no-intercept models having slopes of 13.6 and 15.1, respectively for the C- and X-band SRTM. A lower slope value for C-band than for X-band was contrary to what we expected. The penetration of the C-band microwaves, having slightly longer wavelength, should be some 2–3 m deeper into the canopy than the X-band [36]. This should produce lower height values for C-band as compared to X-band in forested areas. A given biomass value would correspond to a lower C-band than X-band height, and hence a higher biomass-to-height ratio for C-band, which is contrary to what we found here. This unexpected finding must be attributed to random errors in the modelling, e.g. random errors in the GCP points used. The lower spatial resolution and lack of details in the C-band DSM increase the probability of errors in the parameter estimate. The accuracy was similar for the three InSAR data sets, varying from 53.9 to 59.9 t/ha (Table 1).

**Table 1:** **Separate models for each year and each band: Estimating the relationship between AGB and the three alternative InSAR heights, using no-intercept regression models**

InSAR data	Year	Slope, t/ha/m	RMSE, t/ha
SRTM-C	2000	13.6	59.9
SRTM-X	2000	15.1	53.9
Tandem-X	2011	14.9	54.7

By applying these models we obtained AGB changes in alternative ways. The real AGB changes as obtained from repeated field inventory had a mean value of 14.7 t/ha, while the predicted values varied from 12.1 to 21.9 t/ha (Table 2). The major finding here is that the apparent ideal case based on a 30 m resolution X-band DSM in the year 2000, having a DTM, and separately calibrated AGB - InSAR models at both points of time (alternative 4) was only marginally better than the crude case with the 90 m C-band DSM and using only one AGB — InSAR model from Tandem-X data (alternative 1). The bias was of the same magnitude, a few t/ha, although with opposite signs, and the accuracy and correlation was only slightly better with alternative 4. The two alternatives in between, i.e. alternatives 2 and 3, had bias and accuracy values of fairly the same magnitude. Hence, if forest C changes since 2000 are to be estimated with InSAR data, this could be based on the crude alternative 1, which would be the only feasible alternative in many tropical countries. One explanation for bias is the lack of complete synchronicity of the field and the InSAR data, i.e. 8 month difference at the beginning of the period and one year at the end. Real AGB changes due to logging and forest growth may explain some of the bias. For example, if one of the field plots had been logged between the last ALS acquisition and the Tandem-X acquisition, and the InSAR height decreased from 20 to 0 m above ground on this plot, this would generate a bias of -0.11 m when averaged over the 176 field plots. In addition, the results are sensitive to the GCPs used. In this study we placed GCPs subjectively taking into account the differential interferograms. Poorly placed GCPs could generate a bias.

**Table 2:** **Comparison of performance of model alternatives for ΔAGB (t/ha)**

Alt	Description	ΔAGB	Bias	Accuracy	Precision	r
1	90 m C-band SRTM as reference DSM, AGB model calibrated only in 2010	12.1	-2.6	65	65	0.66
2	30 m X-band SRTM as reference DSM, AGB model calibrated only in 2010	20.4	5.7	59	59	0.73
3	90 m C-band SRTM as reference DSM, AGB calibrated with separate models in 2000 and 2010	21.9	7.2	63	63	0.67
4	30 m X-band SRTM as reference DSM, AGB calibrated with separate models in 2000 and 2010	19.0	4.3	59	59	0.73

Field measured ΔAGB was used as the reference with a mean value of 14.7 t/ha.

A particular feature of using surface models for C change monitoring is that it is sensitive to height biases. A tiny bias on height changes over large areas would translate into a considerable bias on the estimated REDD credit. There was a negative bias of 2.6 t/ha on the predicted AGB changes with alternative 1 (Table 2). The predicted mean change on the field plots was 12.1 t/ha while the field measured value was 14.7 t/ha. Although this makes up only 2% of the mean standing AGB in 2010, it represents a 18% underestimation of the change in AGB and the above-ground C stock. In a performance based payment system this would correspond to an underestimated REDD-credit of 2,400 US$ per km^2^, if we assume that C makes up 50% of AGB and that the payment is 5 US$ per ton CO_2_. Based on the 176 field plots the real REDD-credit should have been about 14,000 US$ per km^2^, while the estimated REDD-credit would be about 11,000 US$ per km^2^. This bias corresponds to a -0.17 m bias on the change in InSAR surface height, and this illustrates that a minor bias on the derived DSMs and their changes over time can generate a considerable error if the bias occurs at large scale. However, it is likely that an error like this would vary over the area of interest, i.e. that there would be both negative and positive biases varying smoothly over the landscape due to errors varying from SAR image to SAR image.

### Accuracy in spatial variation of AGB changes

**Figure 3:**
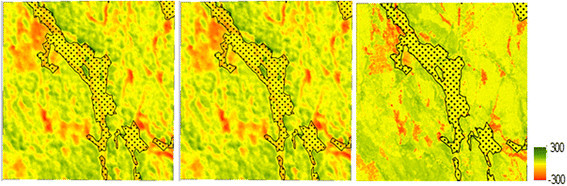
**Predicted AGB change (t/ha) over the study area based on height changes from C-band SRTM to Tandem-X and the model AGB = 14.9IH (left), based on SRTM X-band (middle), and based on repeated ALS acquisitions (right).** Agricultural fields are outlined and marked with black dots.

There was clearly a correspondence in the spatial pattern of AGB changes (Figure 3). Clear-cuts with reduction in AGB and C stocks are visible as red areas, while forest growth representing increases in AGB and C is visible in green. As expected, the ALS data apparently provided more accurate data by having sharp edges on clear-cuts, while the InSAR-based changes were smoother. The changes from X-band SRTM had more distinct change features than those from the more coarse resolution C-band DSM. There were moderately strong correlations between the AGB changes obtained from ALS and those obtained from InSAR. It is notable that the C-band SRTM performed almost equally well as the X-band SRTM. The correlation coefficient was r = 0.62 with the X-band and r = 0.65 with the C-band SRTM (Table 2). ALS is here used as reference data, because it was not feasible to get field measurements covering the entire study area. The real accuracy of the InSAR based changes is unknown. If we assume that the errors on ΔAGB from ALS and errors on ΔAGB from InSAR are uncorrelated, then the spatial variation of InSAR-based changes are more accurate than these results indicate.

It is likely that some loggings occurred between the first ALS acqusition on 8^th^ and 9^th^ June 1999 and the SRTM acquisition in February 2000 (8 months), and between the last ALS acquisition on 2^nd^ July 2010 and the Tandem-X acquisition on 14^th^ July 2011 (1 year). This would include both thinning and clear-felling, and would generate errors in the relationships. The real relationships between changes in biomass and changes in InSAR height are likely to be more accurate than what was obtained here. We had no logging records available and could not quantify the effect of the slight mismatch of the timing of the data acquisitions.

For the entire study area the mean height change for all pixels from C-band SRTM to Tandem-X was a decrease ΔDSM of 0.046 m which corresponded to a predicted mean decrease of ΔAGB = 0.71 t/ha. Estimating the accuracy of this estimate was not within the scope of the present study, and would require further and careful analyses.

We see the presented method as a possible approach to provide MRV data for REDD. First, it can provide complete coverage. This is not a requirement in REDD; however it would enable statistics for various spatial scales. Both the national and sub-national level is defined in REDD, in addition to project areas [37]. This means that an entire country can participate in REDD and receive a REDD credit. However, also a part of a country can be an entity in REDD. This can be an administrative unit such as a state within a federation, it can be a forest type region such as a mangrove forest area, or it can be an area covered by a project addressing forest C stocks. In addition, a given country may want to distribute its REDD credit internally, i.e. setting up a performance based payment system within their country.

Secondly, the method were here tested in a Norwegian forest; however, we believe that the method would be appropriate also in a tropical forest. The two requirements for using the method are that the relationship between AGB and InSAR height (i) is straight linear, and (ii) is stable over time. A straight linear relationship has also been found in a virgin tropical forest in Brazil by [33]. They used the airborne OrbiSAR system, which provided X- and P-band InSAR data. The InSAR height for the forest was derived as the difference between the X- and P-band DSMs. The relationship had a slope (proportionality) of about 13.5 t/ha, which is close to what we have obtained for spruce forests in Norway [24],[25],[38]. However, the straightness of the relationship might vary with forest type. In a regular Eucalyptus plantation in Brazil the relationship was found to be curvilinear [39]. Secondly, the stability of the relationship across weather conditions is also promising. It has been shown in a study in Siberia that frost lowered the L-band InSAR height with 4 m [40]. In a Finnish study by [41], the X-band InSAR height in a pine forest decreased during the autumn, however, that was attributed to needle fall. In a study in Norway we found a considerable decrease in InSAR height for frozen conditions, as compared to non-frozen. However, we found little or no difference between acquisitions during unfrozen conditions spring-summer-autumn [42].

Thirdly, the data requirements should be feasible. Tandem-X has covered the Earth already two times or more, i.e. in 2011 and 2012, and apparently the two satellites will continue to go in Tandem-X formation for some more years with the remaining fuel, which will enable systematic cover of tropical countries in some more years. At the end of the Tandem-X lifetime, a continuation might be covered by best-available-alternatives. BIOMASS is a tailored mission for forest biomass probably operational from 2021 or 2022 [43]. In the meantime we would have to use the best available technology. This could be X-band radargrammetry which can provide almost equally accurate biomass estimates as Tandem-X [44] or optical stereo imaging. Possible new SAR missions in the next years include SAOCOM-CS and Tandem-L. In addition, the SRTM DSM would be invaluable for deriving data on business-as-usual, from 2000 up to Tandem-X in 2011. Field inventory would be necessary to calibrate the relationship between biomass and InSAR height. Such relationships need to be calibrated for various forest types. As indicated by the present study, it is apparently sufficient to do this calibration at one point of time.

In this study we are addressing above-ground biomass only, while an MRV system would require monitoring also of below ground biomass, dead wood, litter and soil. Hence, our proposed InSAR method doesn’t solve all the needs for an MRV system; however, it is a possibly important step in the right direction. Below-ground biomass C can be estimated as it normally makes up an amount proportional to above ground biomass C. Dead wood, litter and soil C are more difficult to estimate, and sophisticated models such as the Yasso model [45] seems to be the only feasible way to derive litter and soil C.

## Conclusions

In conclusion, AGB changes could be estimated fairly accurately from the DSM changes between the SRTM C-band DSM in 2000 and a Tandem-X DSM in 2011. Estimated AGB changes for 200 m^2^ plots (and for 10 m × 10 m pixels) were close to unbiased, with accuracy and precision around 50%. The derived ΔAGB values from InSAR varied consistently with those obtained from ALS over the study area. The performance of the method was slightly better with X-band SRTM data than with C-band. The accuracy was negligibly improved in the case where separate AGB — InSAR height models were fitted for both points of time, which required AGB data for both points of time as well as an accurate DTM.

## Methods

### Study area

The study area was covered by field inventory, ALS and InSAR data at two points of time, i.e. approximately in 2000 and 2011. It was located in a forest area in the municipality of Våler (59°30'N 10°55'E, 70–120 m above sea level), southeast Norway, of about 852.6 ha. The main tree species was Norway spruce (*Picea abies* (L.) Karst.). Scots pine (*Pinus sylvestris* L.) was also quite frequent and there were some scattered broadleaves, in particular birch (*Betula pubescens* Ehrh.). The forest has been actively managed for timber production, including commercial thinning, clear-felling followed by planting in the spruce dominated stands, and selective logging followed by natural regeneration in the pine dominated stands. The study area including the field and ALS data have earlier been used and described in detail by [46] and [47].

### Field plot data

We established 176 circular 200 m^2^ plots for field measurements of above ground biomass. The study area was stratified into four predefined forest types; (1) recently regenerated forest (age ≥ 20 years), (2) young forest, (3) spruce dominated mature forest and (4) pine dominated mature forest. This was done based on an existing stand map. We laid out plots in systematic grids in each stratum. Each plot center was initially located in the field using 1:5000 topographic maps, and their positions were later determined accurately using Differential Global Positioning System (GPS) and Global Navigation Satellite System (GLONASS) having an accuracy of < 0.5 m [46]. The field inventory was carried out in the summers of 1998 and 1999, and then redone in the fall of 2010 and spring of 2011. The field inventory comprised recording of tree species, callipering of all trees with diameter at breast height (dbh) ≥4 cm, and height measurements with a Vertex hypsometer on sample trees selected with a probability proportional to stem basal area. The number of trees with height measurements ranged from 3 to 43 per plot with an average of 18. Non-measured tree heights were later obtained from species specific diameter-height relationships.

AGB was estimated as the sum of the individual components stump, stem, bark, dead and living branches and foliage of individual trees predicted using previously fitted species-specific allometric models having tree species, *dbh* and height as input variables [48]. AGB was estimated for both points of time, from which we also obtained AGB changes at the plot level (Table 3).

**Table 3:** **Above-ground biomass on the plots (t/ha)**

	Mean	min - max
AGB 1999	112	2 - 349
AGB 2010	127	0 - 407
ΔAGB	15	-275 - 153

### ALS data

The study area was covered by complete cover ALS data acquired under leaf-on conditions on 8^th^ - 9^th^ June 1999 and 2^nd^ July 2010 with a Piper PA-31-310 Navajo aircraft at a speed of 70 – 80 m/s at an elevation of 700 – 900 m above the ground (Table 4).

**Table 4:** **Key parameters for the airborne laser scanning campaigns**

Parameter	1999	2010
Instrument	Optech ALTM 1210	Optech ALTM Gemini
Pulse repetition frequency	10 kHz	100 kHz
Scan frequency	21 Hz	55 Hz
Scan half-angle (after processing)	14.0°	13.8°
Pulse density on ground	1.2 m^-2^	7.3 m^-2^

A DTM was derived from the ALS acquisitions, and the heights of the echoes were recalculated to heights above the ground.

Model-predicted AGB changes from the repeated ALS acquisitions were used as a reference representing the true spatial variation of ΔAGB. These ALS-based predictions were produced with separate models for six strata (Table 5). The strata mainly represent the age and the dominating tree species, while one stratum contains stands having thinning during the study period. For each plot we extracted height distribution metrics of the two main ALS echo types (first and last), including height percentiles, cumulative canopy density above certain thresholds, coefficient of variation and mean. We did this for both the 1999 and the 2010 laser scans, and extracted the temporal changes in these metrics. Simple linear models for predicting ΔAGB were developed by regressing field measured ΔAGB against these ALS height metric changes. We applied a stepwise regression model with forward selection of explanatory variables.

**Table 5:** **ΔAGB models based on airborne laser scanning**

Stratum	Model^1^	RMSE, t/ha
1. Clear-cut	ΔAGB = 1.27 + 385 *δdl5*	37.1
2. Thinned	ΔAGB = -78.1 + 14.9 *δpf0* + 26.1 *δpf20* + 3.65 *δcvf* -16.3 *δpl0*	13.3
3. Recently regenerated forest	ΔAGB =2.41 + 17.9 *δpf20*	26.3
4. Young forest	ΔAGB = -56.3 + 32.2 *δmeanf*	36.5
5. Spruce dominated mature forest	ΔAGB = 0.879 -233 *δdf3* + 6.53 *δpl10* + 13.7 *δpl90*	26.4
6. Pine dominated mature forest	ΔAGB =8.28 + 15.1 *δpf50* -89.1*δdf8-7.53 *δpl60* + 8.67 *δpl80*	22.2

^1^p = height percentile of vegetation echoes (0, 10,…, 90); d = cumulative canopy density above vegetation threshold (0, 1, …, 9); cv = coefficient of variation of height of vegetation echoes; mean = arithmetic mean of height of vegetation echoes; f = first echo; l = last echo.

The number of selected metrics varied from one to four. As an example, the model for change in above ground biomass in young stands, stratum 4, was -56.3 + 32.2 *δmeanf*, where *δmeanf* was the change in the mean height of the first echoes. We used these regression models to predict ΔAGB for each 10 m × 10 m pixel over the entire study area. For land cover types other than forest land we applied the model for stratum 1, clear-cut. In order to compare the predicted changes from InSAR and from ALS we selected 784 pixels as a systematic grid over the area.

### InSAR data

**Figure 4:**
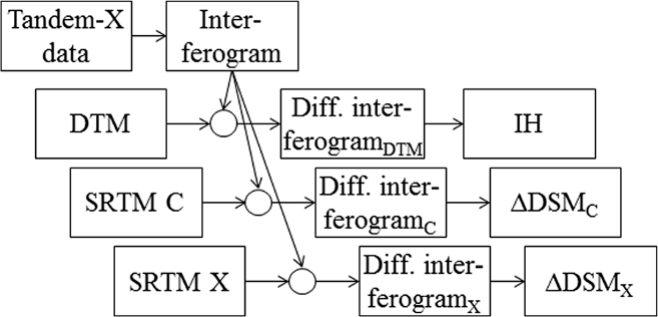
**Overview of the InSAR processing.**

For calibrating the model of AGB against InSAR height we processed the Tandem-X data against the DTM from ALS, while for obtaining the temporal changes in AGB from temporal changes in surface height we processed them against the SRTM DSMs (Figure 4).

Temporal changes in AGB were to be detected as changes from the SRTM DSMs to a Tandem-X DSM, corresponding to changes in canopy height.

We used both the X- and C-band SRTM data, which were acquired during 12^th^ – 20^th^ February 2000. Both the X- and C-band SRTM data have full areal coverage in the study area. We obtained these data as DSMs, i.e. the X-band DSM from the German Aerospace Centre (DLR) in 2002, and the C-band DSM was derived from a procedure built into the ENVI/Sarscape 5.0 software, which downloads data as SRTM-3 version 4 (http://srtm.csi.cgiar.org/index.asp). The data were received in geographic (lat/lon) projection with a spatial resolution of 1 arc sec (15 m x 31 m) for the X-band and 3 arc sec (46 m x 93 m) for the C-band, which we resampled with bilinear interpolation to UTM32 and 10 m x 10 m pixels.

**Figure 5:**
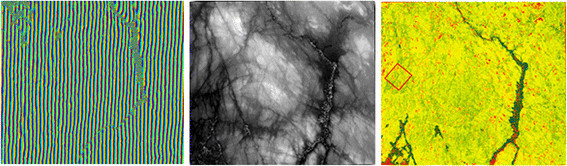
**Processing from interferogram (left) into DSM (middle) and height change from SRTM C-band to Tandem-X (right).** Clear-cuts during the 11 years are clearly visible as red areas. The SAR image covers about 35 km x 35 km. The study area is indicated as a red rectangle.

The Tandem-X data were from an ascending, right looking TanDEM-X stripmap image pair acquired in the morning on 14^th^ July 2011 (Table 6). The data were received from DLR as co-registered Single Look Complex (SLC) data in CoSSC format. The basic idea with InSAR processing is to extract the phase component from the complex data in each of the two SAR images, i.e. to derive the phase difference for each pixel in the form of an interferogram, from which topographic information can be extracted. The InSAR processing was carried out with the ENVI/Sarscape software. The processing was done three times, i.e. separately with the X-band SRTM DSM as a reference, with the C-band SRTM DSM as a reference, and with the ALS DTM as a reference. In each the interferogram was processed into a differential interferogram, being the part of the phase differences in the Tandem-X image pair which represented either the 11 year changes in canopy height (from SRTM) or the canopy height (from DTM). We carried out phase unwrapping using the Minimum Cost Flow method, and converted the unwrapped phases into elevation data and transformed from satellite slant-range geometry to a geocoded DSM. During the processing we used a multi-looking of 5 azimuth x 5 range, which corresponded to about 10 m x 10 m, which was also the spatial resolution of the geocoded DSMs obtained by bilinear interpolation (Figure 5).

**Table 6:** **Technical properties of the Tandem-X InSAR data acquisition, incidence angle θ**_**I**_ **, normal baseline B⊥, and height of ambiguity HoA**

Date	Time	Orbit	Polarization	θ_I_, degrees	B⊥, m	HoA, m
14^th^ July 2011	05:32	Descending	HH-HH	46	55	147

It is important for the accuracy of this method to minimize height errors. In a DSM there is typically one height error which varies from pixel to pixel as random noise. In the processing we applied the Goldstein filter [49] to reduce this noise. In the Tandem-X WorldDEM^TM^ specification this error is quantified as relative vertical accuracy of < 2 m or < 4 m depending on the slope. The second type of error is quantified as absolute vertical accuracy and should be < 10 m. This is a type of error that varies gradually over larger distances, and in the present study area of limited size such an error might appear as a bias or a ramp. In the processing we have largely removed bias and ramp errors by using ground control points (GCPs). We selected points manually in the data where two DSMs were expected to have identical values, i.e. where they could be tied together. They were carefully selected in order to be useful for all DSM corrections, and should represent locations without forests (zero canopy height) and without any temporal changes. This was accomplished by taking into account three differential interferograms, i.e. from the InSAR processing of Tandem-X against the DTM; against the C-band SRTM DSM; and against the X-band SRTM DSM. They were placed in sites having a low fringe density, and where the phase values clearly indicated no canopy height and no temporal change. We fitted Equation (1) to these GCPs:

2 $$\Delta \varphi =k_{0}+k_{1}\;\mathrm{RG}\;+k_{2}\mathrm{AZ}$$

where *Δφ* was the phase difference at each GCP, *k*_*0*_, *k*_*1*_ and *k*_*2*_ were correction factors, and RG and AZ were the range and azimuth co-ordinates (Table 7). After correcting the differential interferogram with these factors the RMSE (root mean square error) of the GCP heights was in the range of 1.3 – 1.5 m. From these corrections we also derived ramp corrections for the SRTM DSMs, which enabled us to extract regression models of AGB against InSAR height for the two SRTM DSMs.

**Table 7:** **Correction factors for phase offset and phase ramp errors (radians), see Equation (** 1 **), and the final accuracy (RMSE) for the 39 Ground Control Points (GCP)**

Reference DSM	***k***_***0***_	***k***_***1***_	***k***_***2***_	RMSE
ALS DTM	0.1603781470	-0.0000644062	0.0000598075	1.54 m
SRTM-C	0.1027309745	-0.0000652833	0.0000286684	1.29 m
SRTM-X	0.2173415607	-0.0000852327	0.0000247047	1.27 m

Weather conditions influence the penetration of SAR microwaves into the vegetation. In dry or frozen conditions the penetration is deeper than in moist conditions because the amount of liquid water in the canopy is low, and the dielectric constant is low [50],[51]. According to this we might expect an InSAR DSM to vary between the acquisitions. Apparently, this has played a minor role in this case, because the obtained relationship between AGB and InSAR height was very stable from the February SRTM acquisitions to the July Tandem-X acquisition. It is possible that this effect is minor in general, or that the dielectric properties were similar although the SRTM was in the winter while the Tandem-X was in the summer. The weather in the study area during 12^th^ – 20^th^ February 2000 was unstable and varied with temperatures around and slightly above zero, and with some precipitation coming as moist snow and sleet. For the Tandem-X acquisition the mean temperature was 14°C and no precipitation from 08.00 13^th^ July – 08.00 14^th^ July.

### Analyses

We derived the relationship between AGB and InSAR height from field inventory on 200 m^2^ plots in fall 2010 and spring 2011, each linked to one 10 m x 10 m InSAR height pixel from the combination of a Tandem-X DSM from July 2011 and a DTM from ALS. This model was based on all field plots without taking tree species into account, because the field plots were dominated by spruce and in most cases contained a mixture of species. Spruce was present in 90% of the plots, and made up 59% of AGB on the plots. We could have estimated species specific models, and they would likely be different [25]. However, the number of plots for each model would then have been small and increasing the random error of the parameter estimates. Similarly, for the temporal changes we fitted a model ΔAGB from field inventory against height changes of the surface models, i.e. ΔDSM. We applied the following statistical measures where *N* is the number of observations, *P*_*i*_ is the predicted value for observation *i*, and *O*_*i*_ is the observed value for observation *i*:

3 $$\mathrm{Bias}=N^{-1}\underset{i}{\Sigma }\left(P_{i}-O_{i}\right)$$

4 $$\mathrm{Accurracy}\;=\mathrm{RMSE}={\left[N^{-1}\underset{i}{\Sigma }{\left(P_{i}-O_{i}\right)}^{2}\right]}^{0.5}$$

5 $$\mathrm{Precision}\;=\mathrm{RMS}E_{s}={\left[N^{-1}\underset{i}{\Sigma }{\left(P_{i}-O_{i}-\mathrm{bias}\right)}^{2}\right]}^{0.5}$$

The evaluation of the spatial accuracy on temporal C changes was based on a visual examination of mapped changes and correlation analyses. Estimated AGB changes from the repeated ALS acquisitions were used as a reference representing the "true" spatial variation of ΔAGB. We are aware that also ALS based changes have errors; however, this was the best available data set for spatial variation in changes. We laid out a 10 m x 10 m grid over the study area and predicted AGB changes for each grid cell with InSAR and ALS. The ΔAGB was predicted directly from the DSM change from SRTM to Tandem-X multiplied by the slope of the relationship between AGB and InSAR height. We selected 784 pixels distributed systematically over the area and extracted the predicted AGB changes from ALS and from InSAR, and used Pearson correlation analyses to represent the spatial correspondence.

## Authors’ contributions

All authors have made substantial contributions and have given final approval of the version to be published. Næsset, Gobakken and Bollandsås have contributed with field and ALS data together with variables derived from them and their documentation, while Solberg has carried out the InSAR processing and done the majority of the writing.

## Authors’ information

All authors have a master degree in forestry, and are working mainly on remote sensing in forestry at a university campus at Ås, Norway. S.S. is a senior researcher working on forest disturbance and remote sensing since 1990 at the Norwegian Forest and Landscape Institute, formerly the Norwegian Forest Research Institute. The three other authors are working closely together mainly on airborne laser scanning in forestry in the forest mensuration group at the Norwegian University of Life Sciences. The group is led by E.N., who is professor and a world leading researcher in the field. T.G. is professor and O.M.B. is a researcher in this group.

## Authors’ original submitted files for images

Below are the links to the authors’ original submitted files for images. Authors’ original file for figure 1 Authors’ original file for figure 2 Authors’ original file for figure 3 Authors’ original file for figure 4 Authors’ original file for figure 5

## Abbreviations

AGB: Above-ground biomass
ALS: Airborne laser scanning
C: Carbon
dbh: Diameter at breast height
DSM: Digital surface model
DTM: Digital terrain model
GCP: Ground control point
IH: InSAR height
InSAR: Interferometric, synthetic aperture RADAR
MODIS: Moderate resolution imaging spectroradiometer
REDD: Reduced Emissions from Deforestation and forest Degradation in Developing Countries
RMSE: Root mean square error
SAR: Synthetic aperture RADAR
SRTM: Shuttle RADAR topography mission
