# Supplementary figures and images for: Forest biomass change estimated from height change in interferometric SAR height models
Source: Carbon Balance Manag. 2014 Sep 10;9:5. doi: 10.1186/s13021-014-0005-2 (PMC4159577; doi:10.1186/s13021-014-0005-2)

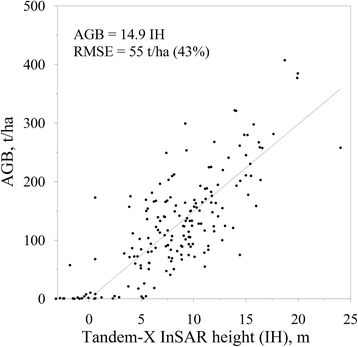

Supplement: Supplementary file 1 — Authors’ original file for figure 1 [file 13021_2014_5_MOESM1_ESM.gif]

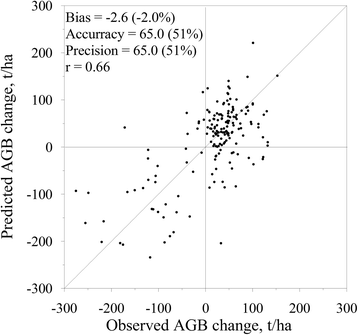

Supplement: Supplementary file 2 — Authors’ original file for figure 2 [file 13021_2014_5_MOESM2_ESM.gif]

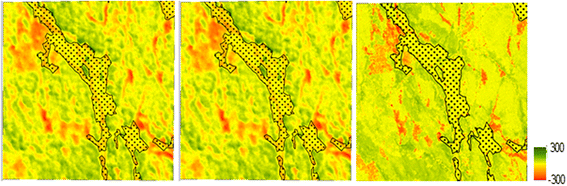

Supplement: Supplementary file 3 — Authors’ original file for figure 3 [file 13021_2014_5_MOESM3_ESM.gif]

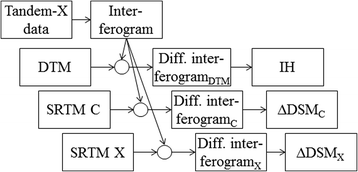

Supplement: Supplementary file 4 — Authors’ original file for figure 4 [file 13021_2014_5_MOESM4_ESM.gif]

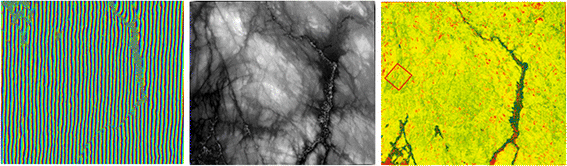

Supplement: Supplementary file 5 — Authors’ original file for figure 5 [file 13021_2014_5_MOESM5_ESM.gif]
